# Demonstration and Assessment of Purification Cascades for the Separation and Valorization of Hemicellulose from Organosolv Beechwood Hydrolyzates

**DOI:** 10.3390/membranes12010082

**Published:** 2022-01-12

**Authors:** Roy Nitzsche, Hendrik Etzold, Marlen Verges, Arne Gröngröft, Matthias Kraume

**Affiliations:** 1 DBFZ Deutsches Biomasseforschungszentrum gemeinnützige GmbH, Torgauer Straße 116, 04347 Leipzig, Germany; hendrik.etzold@dbfz.de (H.E.); arne.groengroeft@dbfz.de (A.G.); 2 Fraunhofer Center for Chemical-Biotechnological Processes (CBP), Am Haupttor-Bau 1251, 06237 Leuna, Germany; marlen.verges@igb.fraunhofer.de; 3 Chemical and Process Engineering, Technical University Berlin, Marchstraße 23, 10587 Berlin, Germany; matthias.kraume@tu-berlin.de

**Keywords:** lignocellulose biorefinery, hemicellulose, xylose, hydrothermal treatment, nanofiltration, adsorption, ultrafiltration, flowsheet simulation, costing

## Abstract

Hemicellulose and its derivatives have a high potential to replace fossil-based materials in various high-value-added products. Within this study, two purification cascades for the separation and valorization of hemicellulose and its derived monomeric sugars from organosolv beechwood hydrolyzates (BWHs) were experimentally demonstrated and assessed. Purification cascade 1 included hydrothermal treatment for converting remaining hemicellulose oligomers to xylose and the purification of the xylose by nanofiltration. Purification cascade 2 included the removal of lignin by adsorption, followed by ultrafiltration for the separation and concentration of hemicellulose. Based on the findings of the experimental work, both cascades were simulated on an industrial scale using Aspen Plus^®^. In purification cascade 1, 63% of the oligomeric hemicellulose was hydrothermally converted to xylose and purified by nanofiltration to 7.8 t/h of a xylose solution with a concentration of 200 g/L. In purification cascade 2, 80% of the lignin was removed by adsorption, and 7.6 t/h of a purified hemicellulose solution with a concentration of 200 g/L was obtained using ultrafiltration. The energy efficiency of the cascades was 59% and 26%, respectively. Furthermore, the estimation of specific production costs showed that xylose can be recovered from BWH at the cost of 73.7 EUR/t and hemicellulose at 135.1 EUR/t.

## 1. Introduction

The strong global dependence on fossil fuels results from the intensive use and consumption of petroleum and natural-gas-based derivatives [1]. With the risks of diminishing reserves and growing climate change, a progressive shift from fossil-based chemistry to bio-based chemistry is necessary to ensure the long-term supply of chemicals, fuels, and materials. The use of integrative and multifunctional biorefineries in which lignocellulosic raw material, e.g., straw or wood, can be fractionated in its three main constituents, cellulose, hemicellulose, and lignin, is seen as a path with excellent prospects in future bioeconomies [2]. In Germany, beechwood was identified as a promising feedstock for lignocellulose biorefineries (LCBs). Accounting for 15% of total forestland and 17% of total wood reserves, beech is the third most common tree species and has an unused potential of 1.3 Mt of dry matter per year [3].

**Figure 1:**
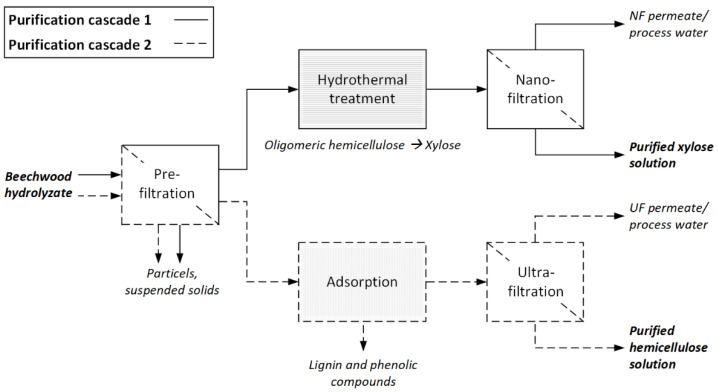
Purification cascades 1 (solid line) and 2 (dotted line) for the separation and valorization of hemicellulose from organosolv beechwood hydrolyzate.

Within a LCB, size reduction and a pulping process realize the breakdown of the beechwood structure. The organosolv process, i.e., pulping with ethanol–water, benefits from relatively mild process conditions and easy-to-recover solvents [4]. The released cellulose can be used to produce pulp, which is processed to boards, fibers, and paper or hydrolyzed to sugars [5]. Lignin can directly be used as a substitute in binding agents and fillings or depolymerized into smaller molecules [6]. The hemicellulose oligomers and their degradation products (sugars, furans, and carboxylic acids) end up in the so-called beechwood hydrolyzate (BWH) [7]. Due to relatively low concentrations, inhomogeneous composition, and the presence of plenty of impurities, the components in BWH are most challenging to recover for further use [8]. However, several studies have shown that if the hemicellulose and its derived sugars are separated from the BWH, they could be used to produce high-value-added products such as renewable hydrogels [9], barrier films [10,11], paper additives [12,13], coatings [13], and platform chemicals such as xylitol, furfural, lactic acid, and levulinic acid [14,15]. Hemicellulose is required for the first four product classes, whereas monomeric sugars serve as the starting material for the production of platform chemicals. Since both hemicellulose and monomeric sugars are of great interest for bio-based chemistry [16,17], for each component, a purification cascade for its provision and, thus, valorization from organosolv BWH (Figure 1) was experimentally developed and technically and economically assessed.

Purification cascade 1 consists of hydrothermal treatment of the BWH to hydrolyze the remaining hemicellulose oligomers into monomeric C5 sugars and nanofiltration (NF) to separate potential fermentation inhibitors (e.g., carboxylic acids and furanic compounds) and increase the concentration of sugars. Various studies have already shown that the hydrothermal conversion of the hemicellulose oligomers into monomeric sugars is a process with good prospects [18,19,20,21]. One of the main challenges during the hydrothermal treatment of biomass-derived hydrolyzate streams is the formation of humins by polymerization reactions and other impurities, such as furanic and phenolic compounds [22,23]. These components can block the reactor system or cause problems in the following processes, such as increased fouling or inhibition. The separation and purification of the monomeric C5 sugars are commonly realized by liming and carbonization processes to remove non-sugars and multi-stage evaporation for concentration [24,25]. However, membrane filtration offers the advantage of removing water and other non-sugars without adding chemicals and reducing process energy since there are no phase changes. A drawback of this process is the potential fouling of the membranes used, which can result in a considerable loss in filtration performance [26,27,28]. Zhou et al. [29] proposed using reverse osmosis membranes to separate fermentation inhibitors from monomeric sugars out of a model solution. On the other hand, Gautam and Menkhaus [26] showed that when applying real biomass streams, NF is recommended due to lower rejections of inhibitors and, hence, less fouling caused by osmotic pressure. This was confirmed in numerous other studies [21,30,31,32,33].

Purification cascade 2 consists of an adsorption process to remove foulants such as lignin from the BWH and ultrafiltration (UF) for the concentration of hemicellulose and simultaneous removal of remaining lignin fragments and smaller molecules. The treatment of wood hydrolyzates with adsorption on activated carbon, zeolites, and polymeric resins has been proven to be effective in removing lignin, its degradation products, and furanic compounds [34,35,36,37,38,39]. However, the loss of hemicellulose during this treatment can be significant. To minimize this, adsorption materials with a high selectivity must be identified. Moreover, the adsorbent should be easy to regenerate (e.g., desorption by solvent washing with ethanol [34]) without appreciable adsorbent losses. With regard to these requirements, polymeric resins seem to be promising [34,35]. Furthermore, it has been shown that pretreatment of wood hydrolyzate by adsorption results in higher permeate fluxes in a subsequent UF step due to the removal of hydrophobic components [39,40]. This effect is applied to enhance the filtration capacity during the recovery of hemicellulose using UF. Compared to the commonly used method for hemicellulose isolation, including precipitation and filtration with organic solvents [41,42], additional mass streams and energy can be saved. Various studies have already proven the feasibility of the UF of hemicellulose [40,43,44,45,46].

Although the individual processes have already been investigated, their integration into overall process chains for the valorization of hemicellulose from organosolv BWH has, to the best of our knowledge, not yet been examined. Thus, this study aims to demonstrate and assess both purification cascades (Figure 1) at a laboratory to pilot scale. Apart from that, open issues in the individual process steps were addressed: temperature and pH were varied for the hydrothermal treatment to maximize the oligomeric hemicellulose-to-xylose conversion rate and minimize the formation of chemical successors. A parameter screening for transmembrane pressure and temperature was carried out to achieve an energetically favorable NF process with high xylose recovery rates. Adsorption was designed in terms of a defined benchmark for lignin removal and hemicellulose recovery. The specific influence of adsorption pretreatment on UF concerning permeate flux and retentions was investigated. Finally, mass and energy balances for both purification cascades were calculated using Aspen Plus^®^, and specific production costs were calculated. The results will contribute to expanding their application in real biomass substrates.

## 2. Materials and Methods

### 2.1. Beechwood Hydrolyzates

BWH was provided by the lignocellulose biorefinery pilot plant of Fraunhofer Center for Chemical–Biotechnological Processes (Leuna, Germany). During the investigations for this study, the organosolv process for the pulping of beechwood into cellulose, hemicellulose, and lignin was further developed and optimized. Hence, for the experimental work, BWH from different batches with different concentrations was used. In the following, the varying process conditions for the extraction of the BWHs are described.

During organosolv pulping, 70 kg oven dry weight (odw) of debarked beechwood chips (*Fagus sylvatica*) were fractionated in a 500 L batch reactor with forced circulation at 170 °C and 2.0 MPa for 130 min (H-factor = 1500) using 224 kg of an ethanol–water mixture (mass ratio 1:1) and 0.8% sulfuric acid (based on wood odw) as a catalyst. Afterward, the spent liquor (SL) was displaced by fresh ethanol–water wash liquor (WL, mass ratio 1:1) and washed at 100 °C. Subsequently, two more displacement washing steps were performed with water. The remaining pulp fraction was discharged and dewatered using a screw press. The displaced SL and WL were further processed in a lignin precipitation step. A share of approximately 90% of the dissolved lignin in the SL was precipitated by the evaporation of ethanol at reduced pressure [47] and recovered by filtration. From the remaining liquid phase, ethanol was recovered by rectification, and the residual liquid product is considered BWH. It contains oligomeric hemicellulose and its hydrolysis products, such as monomeric sugars, furans, and carboxylic acids as well as residual lignin and its phenolic derivatives.

In this study, BWH was taken from three different batches, which differ in their proportion of SL to WL. The concentrations of key components of the BWHs were analyzed, and their molar masses and pK_a_ values were taken from the literature (Table 1). BWH 1 and 2 are used for the experimental investigations for purification cascades 1 and 2, respectively. The composition of BWH 3 is taken as the assumed feedstock for the process assessment of purification cascades 1 and 2 (see Section 2.5). Before utilizing BWHs 1 and 2, they were filtered by vacuum filtration using 4 μm filter paper (Macherey-Nagel, Düren, Germany) to remove larger particles and suspended solids from the liquid fraction. Afterward, they were stored at 4 °C up to the utilization. The pH of BWH 1 and 2 is 2.6 and 1.9, respectively. Using a 5 M NaOH (VWR Chemicals, Darmstadt, Germany, purity ≥98.9%) solution, the pH of BWH 2 was adjusted to 2.6 as well.

### 2.2. Purification Cascade 1

The following Section 2.2.1 and Section 2.2.2, describe the materials used, experimental setup, and experimental procedure for the hydrothermal treatment and the NF process.

#### 2.2.1. Hydrothermal Treatment

**Figure 2:**
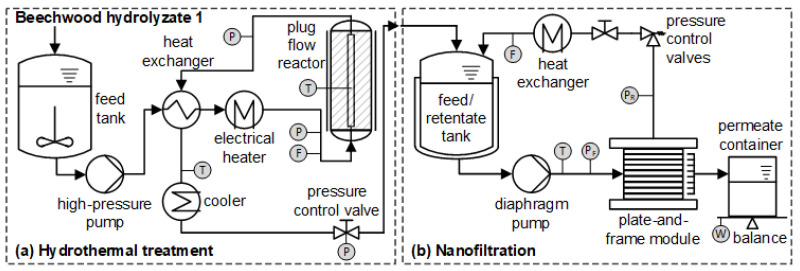
Schematic diagram of the experimental setup for (**a**) hydrothermal treatment and (**b**) nanofiltration.

A continuous process on a pilot scale was developed for the hydrothermal conversion of remaining oligomeric hemicellulose in BWH 1 to monomeric xylose. The experiments were carried out in a plug flow reactor made of Inconel 625 high-grade steel with a total length of 2150 mm, an internal diameter of 40 mm, and a reaction volume of about 2.15 L. In a previous study [21], it was demonstrated that short residence times (3.1 min) at elevated temperatures (180 °C) favor the conversion of oligomeric hemicellulose to xylose. The reactor was fed at a flow rate of 20 L/h by a high-pressure pump to realize the shortest possible average residence time of 9 min. The pressure was adjusted between 12.9 and 16.2 MPa by an automatic adjustable pressure retention valve. The temperature was kept between 174 and 180 °C. In addition, the influence of the pH on the hydrothermal process was determined by lowering it from 2.6 to 1.7 using sulfuric acid (96%). The start-up was done with de-ionized water (DIW), and once the desired parameters were reached, the feed was switched to BWH 1. At the reactor outlet, the reaction product passed through a double pipe heat exchanger, preheating the feed solution to approx. 130 °C and cooling down the reactor outlet to approx. 60 °C. After the heat recovery, the hydrothermally treated BWH 1 was water-cooled by a double pipe heat exchanger, depressurized to ambient conditions, and collected in an intermediate bulk container. Samples of the product streams were taken. A schematic diagram of the experimental setup is shown in Figure 2a.

#### 2.2.2. Nanofiltration

Apart from the xylose, the hydrothermally treated BWH 1 contained components such as furans, including 5-hydroxymethylfurfural (5-HMF) and furfural, and acetic acid, which can have strong inhibitory effects in subsequent fermentation processes. Using a cross-flow filtration system, NF was investigated for the concentration of xylose with simultaneous removal of inhibitors. The polymeric flat sheet membrane NF, obtained from Alfa Laval (Lund, Sweden), was used. It is made of thin-film composite polyamide and has a molecular weight cut-off of 150–300 Da.

The cross-flow filtration system consists of a plate-and-frame membrane module, a 20 L double-jacket feed tank, a diaphragm pump, and a shell and tube heat exchanger (Figure 2b). The active membrane surface area amounts to 0.036 m². The transmembrane pressure (Δp), calculated according to Equation (1), was adjusted by pressure control valves installed on the retentate side and measured by pressure sensors installed on the feed (p_f_) and the retentate (p_r_) side (atmospheric pressure at the permeate side).

(1) $$\Delta p=\frac{p_{f}+p_{r}}{2}$$

The cross-flow was regulated by the pump equipped with a frequency converter and measured on the retentate side. The temperature was controlled by a thermostat connected to the heat exchanger and measured by a Pt100 element behind the pump. The permeate flux (J) through the membrane was determined with a volumetric cylinder placed on an electronic balance and calculated according to Equation (2).

(2) $$J=\frac{V_{p}}{A_{m}\times t}$$

where V_p_ is the permeate volume, A_m_ is the membrane surface area, and t is time.

Before the NF experiments, the membrane was rinsed with DIW and then with a 1 wt.% P3-Ultrasil 53 solution for 30 min at 0.4 MPa and 40 °C. Afterward, the detergent was replaced by DIW, and the NF membrane was compacted. A fresh membrane was used in each trial. For parameter screening studies, 10 L of feed solution were filled into the feed tank and operated in total reflux mode. This means both retentate and permeate were recycled to the feed tank. The transmembrane pressure was varied between 1 and 4 MPa, and the temperature was varied between 25 and 55 °C at a constant flow rate of 14 L/min, which corresponds to a cross-flow velocity of 1.5 m/s and a Reynolds number of 1130. The hydrothermally treated BWH 1 was concentrated under appropriate process conditions to a volume reduction (VR) of 0.8. Small amounts of samples (10 mL) were taken from the feed (f), retentate, and permeate (p) for the determination of the component concentrations (C_i_) and the calculation of observed retentions (R_obs_) according to Equation (3).

(3) $$R_{i,\mathrm{obs}}=\left(1-\frac{C_{i,p}}{C_{i,f}}\right)\times 100\%$$

### 2.3. Purification Cascade 2

The following Section 2.3.1 and Section 2.3.2, describe the materials used, experimental setup, and experimental procedure for the adsorption and UF processes.

#### 2.3.1. Adsorption

Removal of lignin and its phenolic degradation products from BWH 2 by adsorption was investigated in a fixed-bed column. Based on previous findings, the hydrophobic styrene–divinylbenzene-based resin SEPABEADS SP700 (Mitsubishi Chemical Corporation, Düsseldorf, Germany) was used as an adsorbent [35]. Before the adsorption experiments, the resin was rinsed with DIW to remove preservatives such as sodium chloride and sodium carbonate salts and other contaminants. Afterward, it was dried for 24 h at 80 °C. For each experiment, a fresh adsorbent was used.

**Figure 3:**
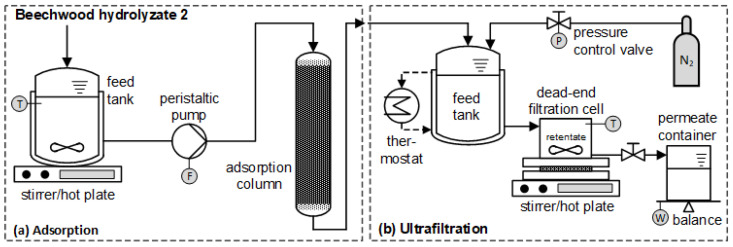
Schematic diagram of the experimental setup for (**a**) adsorption and (**b**) ultrafiltration.

The experimental setup consisted of a 1 L round bottom beaker that was placed in a water bath and agitated with a magnetic stirrer at 500 rpm, a peristaltic pump, and an insulated glass column with a length of 20 cm and an inner diameter of 1.5 cm (Figure 3a). The beaker was covered throughout the trials. Experiments were carried out with a flow rate of 2 mL/min, which corresponds to 4.5 bed volumes (BVs) per hour (recommended by the manufacturer), and a temperature of 50 °C, which was controlled by a Pt100 element inserted in the beaker. The volume of the adsorbent bed was 26.5 mL (h_bed_ = 15 cm; d_bed_ = 1.5 cm), and SP700 was packed to the column as an aqueous slurry. To determine the maximum amount of lignin that can be adsorbed onto SP700, 30 BV of BWH 2 were fed through the adsorbent bed. Each BV was collected in a volumetric cylinder and taken as a sample for analysis.

#### 2.3.2. Ultrafiltration

Isolation and concentration of hemicellulose from the adsorption-treated BWH 2 were conducted by UF using a dead-end filtration system. The polymeric flat-sheet membrane TriSep UA60, obtained from Mann+Hummel (Wiesbaden, Germany), was used in all experiments. UA60 is considered a tight UF membrane; it is made of polypiperazine amide and has a molecular weight cut-off of 1000–3500 Da. The membrane was pretreated with a 1 wt.% P3-Ultrasil 53 solution for 4 h. Afterward, it was rinsed and soaked with DIW for at least 24 h until used in experiments. A fresh membrane was used in each trial.

The dead-end filtration system with a stationary mixer consisted of a 1 L double-jacket feed tank and a 0.4 L stirred cell pressurized with nitrogen (Figure 3b). The active membrane surface area is 0.0044 m². The transmembrane pressure was adjusted by a pressure control valve and measured by a pressure sensor. A stirrer/hot plate was used to set the desired stirring speed and temperature within the stirred cell. In addition, the temperature of the feed tank was measured with a Pt100 element and maintained constant by a water circulation thermostat. The permeate flux through the membrane was obtained by collecting permeate over time in a volumetric cylinder placed on an electronic balance.

To determine the influence of the adsorption pretreatment on the UF process, both untreated and adsorption-treated BWH 2 were tested. Before the filtration experiments, pure water flux (PWF_b_) was measured using DIW. Afterward, 600 mL of the respective feed solution were filled into the feed tank/stirred cell and concentrated to a VR of 0.8. Samples were collected from the feed and permeate to determine the component concentrations and the calculation of observed retentions. Finally, the pure water flux was measured again after the filtration (PWF_a_) in order to assess the pure water flux reduction (PWF_r_), according to Equation (4), as an indicator for the severity of fouling.

(4) $${\mathrm{PWF}}_{r}=\frac{{\mathrm{PWF}}_{b}-{\mathrm{PWF}}_{a}}{{\mathrm{PWF}}_{b}}\times 100\%$$

All filtration steps were operated at a transmembrane pressure of 0.95 MPa, a temperature of 55 °C, and a stirring speed of 500 rpm. The applied process parameters, as well as the UF membrane used, were chosen based on the findings of Nitzsche et al. [46].

### 2.4. Analytical Methods

#### 2.4.1. High-Performance Liquid Chromatography

The concentrations of oligomeric hemicellulose, glucose, xylose, 5-HMF, and furfural in the hydrothermally treated BWH 1 were determined by a 1260 Infinity high-performance liquid chromatography (HPLC) system (Agilent Technologies, Santa Clara, CA, USA) and, for the other investigations, with an AZURA HPLC system (Knauer, Berlin, Germany). Both systems are equipped with a binary pump system, an autosampler, a column oven, a diode array detector (DAD), and a refractive index detector (RID). The autosampler injected 10/15 μL of the sample into an HPX-87H/MetaCarb 87P column (300 mm × 7.8 mm) equipped with a pre-column (30 mm × 7.8 mm) operated at 65/70 °C; 5 mM sulfuric acid/ultrapure water was used as mobile phase under isocratic conditions and a flow rate of 0.6/0.35 mL/min. Glucose and xylose were detected using the RID, and 5-HMF and furfural were quantified with the DAD set at 280 nm. The concentration of oligomeric hemicellulose was determined as a concentration difference of xylose concentration after total hydrolysis. Preparation of the samples and the total hydrolysis have already been described in a previous study [46]. All HPLC measurements were conducted at least in duplicate.

#### 2.4.2. Gas Chromatography

Acetic acid analysis was performed on a 7890 A gas chromatography (GC) system (Agilent Technologies, Santa Clara, CA, USA) equipped with a DB-FFAP column (60 mm × 0.25 mm × 0.5 µm) and a flame ionization detector (FID). For the analysis, 3 mL of sample, 1 mL of internal standard (2-methyl butyric acid, 184 mg/L), 0.5 mL methanol, and 2.5 mL sulphuric acid (diluted 1:5 *v*/*v*) were placed in a headspace vial and closed with an aluminum crimp cap with PTFE/silicone septum. All GC measurements were conducted at least in triplicate. Detailed information about the parameters of the GC system has been published elsewhere [21].

#### 2.4.3. UV/VIS Spectroscopy

For the analysis of lignin concentration, a UV/VIS spectrophotometer (UV-6300PC, VWR Chemicals, Darmstadt, Germany) at a wavelength of 280 nm was used [40,51]. It has to be pointed out that some wood extractives and carbohydrate degradation products (e.g., furfural and 5-HMF) also absorb UV light at 280 nm [34]. This can lead to an overestimation of the lignin concentration. The samples were analyzed at least in duplicate. Further details of the UV/VIS method and the conversion of the obtained absorption rate into a mass concentration have already been published by Nitzsche et al. [46].

### 2.5. Process Assessment

#### 2.5.1. Flowsheet Simulation

Flowsheet simulation using Aspen Plus^®^ (V10, Aspen Technology, Bedford, MA, USA) was chosen to calculate mass and energy balances, energy efficiencies, and the sizing of the equipment. The input data of the flowsheet simulations are from the experimental results, complemented by information from literature, as indicated. Liquid activity coefficients were calculated with the NRTL (Non-Random Two Liquids) property method because of its good performance in representing highly non-ideal mixtures [52]. All unit operations were simulated as continuous processes. The physical property database *BIODFMS3* [53] was used for the physical properties of the main lignocellulosic components (e.g., cellulose, hemicellulose, lignin) [54].

Both purification cascades were scaled to the same plant size and input concentration. A previous study presented an organosolv process, converting 50,000 dry metric tons of beechwood annually, resulting in 258,400 metric tons of BWH [55]. This dimension was found to be reasonable for a demonstration plant [56,57]. Furthermore, the input concentration corresponds to BWH 3, and an output concentration of 200 g/L of the desired components seems attainable [58]. Moreover, it was assumed that the processes would be located in Germany at an existing chemical site, where all required utilities and wastewater treatment could be provided, and the operating time would be 8000 h per year. The utilities needed for the process configurations are high-pressure steam (HPS) at 2.2 MPa, low-pressure steam (LPS) at 0.23 MPa, cool water (CW) with an inlet temperature of 20 °C, and electricity (EE).

#### 2.5.2. Costing

The mass and energy balances from the flowsheet simulation of both purification cascades as well as the sizing of the equipment were used to conduct the costing. Specific production costs were calculated using a simplified approach based on VDI Guideline 6025 [59]. All relevant costs were assigned to the cost group’s capital-linked costs, consumption-linked costs, operation-linked costs, and other costs. The capital-linked costs were determined based on equipment costs. The design, dimensioning, and quantitative determination of the equipment were carried out in the process simulation. The estimation of equipment costs by dimension and material was done according to the data and methods described by Peters et al. [60] and Chauvel [61]. The Chemical Engineering Plant Cost Index (CEPCI) and ProcessNet Chemieanlagen-Index Deutschland (PCD) [62] were used as cost indices to update the costs to the base year 2020. Plant-specific surcharge factors were used to account for direct costs and indirect costs leading to fixed-capital investments (FCIs). The annuity method was used to calculate depreciation and interest into equal annual payments. The consumption-linked costs are based on the use and prices of raw materials, auxiliary materials, process energy, and disposal costs. Operation-linked costs contain estimations for labor and maintenance. Operating staff was assumed based on typical requirements in the chemical industry according to plant capacity and the degree of automation [60]. The wage costs were calculated on an hourly basis [63]. Operating supervision was estimated with a 15% surcharge based on the operating staff cost. Other costs include administration, insurance, and uncertainties, which were calculated with overhead rates. General assumptions for the costing are summarized in Table 2. Specific production costs were calculated by dividing the total annual costs of production by the annual quantity of products.

## 3. Results and Discussion

### 3.1. Purification Cascade 1

#### 3.1.1. Hydrothermal Treatment

Compositions of the hydrothermally treated BWH (HTBWH) 1 are summarized in Table 3. The treatment of HTBWH 1.1 at the original pH of 2.6 and a process temperature of 180 °C resulted in a hydrolytic splitting of 63% of the hemicellulose oligomers and a xylose concentration increase by 37%. Furfural formation was observed to a low extent, proving low sugar degradation. However, to achieve complete hydrolysis, the pH had to be adjusted by adding sulfuric acid since it is acid-catalyzed. At a pH of 1.7 for HTBWHs 1.2 and 1.3, complete hydrolysis of the oligomeric hemicellulose could be observed. Moreover, the results of HTBWHs 1.2 and 1.3 indicate that a decrease in temperature from 180 to 174 °C results in less sugar degradation and, thus, higher xylose concentration. As negative side effects, an increased formation of solids due to polymerization reactions [20] was observed, and the more acidic conditions caused corrosion issues in the plant.

Since the addition of chemicals to BWH 1 is unfavorable from an economic and environmental point of view and due to the material damage from corrosion, a lower xylose yield was accepted, and experiments continued with HTBWH 1.1.

#### 3.1.2. Nanofiltration

**Figure 4:**
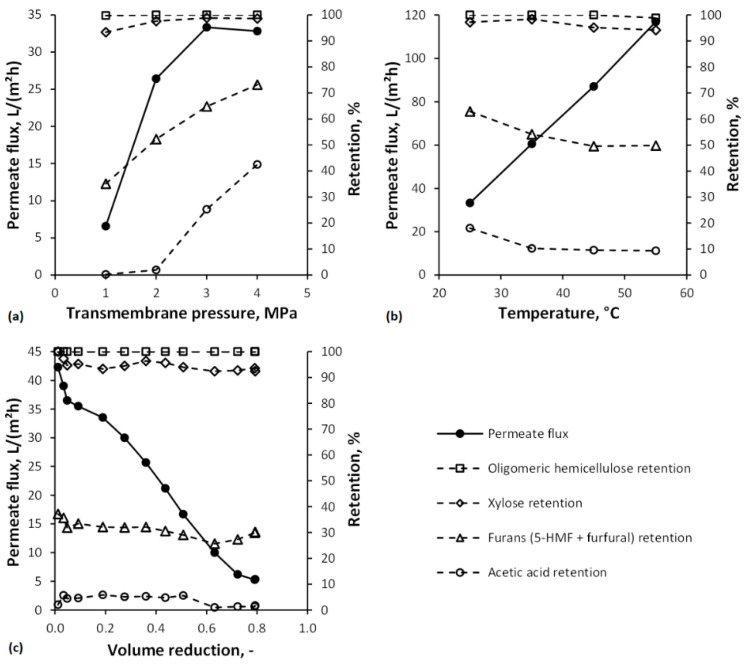
(**a**) Influence of transmembrane pressure on permeate flux and retentions (T = 25 °C), (**b**) influence of temperature on permeate flux and retentions (Δp = 3 MPa), and (**c**) impact of the concentration of hydrothermally treated beechwood hydrolyzate 1 on permeate flux and retentions (Δp = 2 MPa, T = 35 °C).

In Figure 4a, screening experiments with transmembrane pressures of 1 to 4 MPa and their influence on permeate flux and retention are shown. Typically, the permeate flux increases with increasing transmembrane pressure linearly until a certain level, the so-called critical flux [64]. Beyond this critical flux, no further significant increase can be achieved. The critical flux was determined to lie between 2 and 3 MPa. The deviation from the linear increase of permeate flux with transmembrane pressures after 2 MPa indicates the transition to the limiting flux phase. The cause for the flux limitation may be concentration polarization and gel/cake layer formation [65]. This flux limitation cannot be avoided using real wood hydrolyzates. Still, the choice of a fitting transmembrane pressure value permits that the mass accumulation on the membrane surface has a minor effect on the process efficiency. In general, the retentions of the solutes increased as the transmembrane pressure increased. For furans (5-HMF + furfural) and acetic acid, the increase was more significant than for xylose and even oligomeric hemicellulose. The reasons might be an increased solvent flux due to increased pressure since water is more permeable than the solutes and the formation of a fouling layer that acts as an additional permeation barrier and, thus, increases solute rejection [46,66].

The influence of temperature on permeate flux and solute retentions is shown in Figure 4b. The permeate flux increases linearly within the studied temperature range. This trend can be explained by a declining viscosity of the feed solution, which enhances the liquid flow through the membrane and, thus, the permeate flux according to the proportionality $J\approx 1/\eta \approx 1/e^{1/T}$. The decrease in solute retentions can be attributed to several mechanisms: (i) increased diffusion of the solutes, resulting in an enhanced transport through the membrane, (ii) Nilsson et al. [67] proposed that the increase in the mass transfer of uncharged solutes is more significant than that of water with increasing temperature, and (iii) increased temperature can cause changes in the surface structure of thin-film composite membranes, leading to an increment of membrane pore size and molecular weight cut-off [68].

Appropriate process conditions chosen on the beforehand parameter screening were a transmembrane pressure of 2 MPa and a temperature of 35 °C. HTBWH 1.1 was concentrated by NF to a VR of 0.8. Results of the concentration experiments are depicted in Figure 4c. Permeate flux started with 42.3 L/(m²h) and decreased continuously since the feed became more concentrated due to rejected solutes, reaching a value of 5.3 L/(m²h). The average permeate flux was 22.5 L/(m^2^h). The course of the permeate flux indicates severe membrane fouling. As discussed in a previous study, the main reasons for fouling might be due to concentration polarization and the formation of a gel/cake layer [21]. Alkaline cleaning using a 1 wt.% P3-Ultrasil 53 solution was sufficient to remove the foulants and maintain a stable pure water flux. Average retentions for oligomeric hemicellulose, xylose, furans, and acetic acid were 100%, 95%, 31%, and 4%, respectively.

### 3.2. Purification Cascade 2

#### 3.2.1. Adsorption

**Figure 5:**
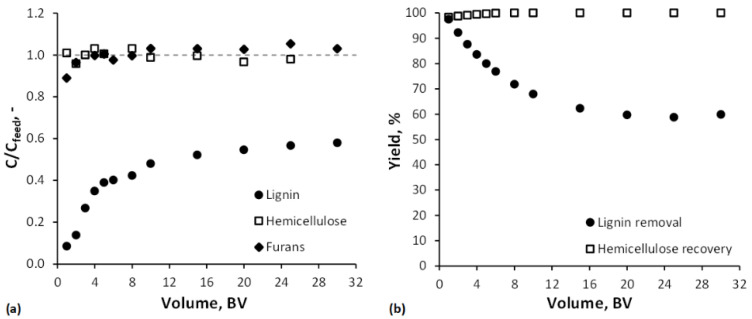
(**a**) Breakthrough curves of lignin, hemicellulose (oligomeric hemicellulose + monomeric xylose), and furans (5-HMF + furfural) and (**b**) lignin removal and hemicellulose recovery as a function of the bed volumes fed through the SP700 adsorbent bed with 4.5 BV/h.

The removal of lignin, hemicellulose (oligomeric hemicellulose + monomeric xylose), and furans (5-HMF + furfural) during the adsorption in a SP700 fixed-bed column is represented by its breakthrough curves in Figure 5a. The breakthrough curve of lignin showed a prolonged and atypical shape. Montané et al. [69] and Heinonen et al. [34] have described similar behavior during lignin adsorption on activated carbon and the resin Amberlite^®^ XAD-16N, respectively. The reason might be the heterogeneous nature of the lignin, meaning it consists of a wide variety of fragments of different molecular sizes and shapes [70,71], eluting in irregular intervals. After feeding 30 BVs of BWH 2 through the SP700 column, the outlet lignin concentration did not reach the feed level. The hemicellulose concentration increased to the feed level within the first BV. Hence, there is no or just minor adsorption on SP700. The course of the breakthrough curve between BVs 0 and 1 could not be determined due to dead volumes in the experimental setup. For the furans, the breakthrough was observed just a few BVs after the hemicellulose. The feed concentration level for the furans was reached after 3 BVs. Due to their hydrophobic nature, the adsorbent material was expected to be more selective regarding these components. Furans and lignin probably compete during the adsorption, and the higher concentration of lignin in BWH 2 could cause the furans to be displaced from the solid phase.

The yield of the adsorption process was calculated in terms of lignin removal and hemicellulose recovery as a function of the BVs fed through the column (Figure 5b). Lignin removal of at least 80% was defined as a benchmark. This was achieved after adding 5 BVs of BWH 2 to the adsorbent bed and is associated with a hemicellulose recovery of 99%.

#### 3.2.2. Ultrafiltration

**Figure 6:**
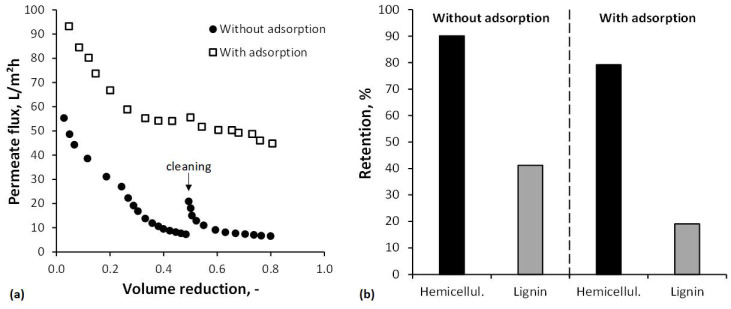
(**a**) Permeate flux and (**b**) retention for hemicellulose (oligomeric hemicellulose + monomeric xylose) and lignin during the ultrafiltration of Beechwood Hydrolyzate 2 with and without prior adsorption using the polymeric resin SP700.

Adsorption previous to the UF process was employed to remove hydrophobic components, mainly lignin, and thus reduce fouling of the UA60 membrane. Figure 6a presents the permeate flux for the filtration of untreated and adsorption-pretreated BWH 2. Pretreatment with polymeric resins significantly increased the permeate flux from an average value of 22.2 to 60.8 L/(m^2^h). This is at least partly due to the removal of lignin and its degradation products, which have been shown to foul the membrane [72]. Another explanation for the increased flux values is provided by Puro et al. [73]: wood extractives, which might be preferentially attached on the membrane surface in the absence of lignin, have both hydrophobic and hydrophilic moieties and, when the hydrophobic part is attached on the membrane, the hydrophilic part faces the aqueous phase, making the membrane surface more hydrophilic and, thus, improving the permeate flux. However, fouling caused by the filtration of BWH 2 was quantified by the calculation of the PWF_r_. Without adsorption before the UF, the PWF_r_ was 27% and, with adsorption before the UF, 8%. Hence, adsorption as a pretreatment for the UF of wood hydrolyzates minimizes membrane fouling. Koivula et al. [40] and Persson et al. [39] made similar findings.

Figure 6b shows the influence of the pretreatment by adsorption on the retention of hemicellulose and lignin. Adsorption reduced the average retention from 90 to 79% for hemicellulose and from 41 to 19% for lignin. This means higher losses of hemicellulose but, simultaneously, a purer retentate stream. Reasons might be reduced interactions between hydrophobic lignin and other unidentified components with the UF membrane and, thus, less formation of a deposited layer and less pore plugging or narrowing.

### 3.3. Process Assessment

For the process assessment, the results of mass and energy balances as well as specific production costs are presented below. Based on the results, both purification cascades are compared with each other. The aim is to identify the purification cascade that is more efficient and cost-effective under the assumed conditions. However, it should be noted that the intermediate products obtained, mainly xylose and hemicellulose, have different values and are, therefore, not directly comparable.

#### 3.3.1. Mass Balance

The input and output mass streams of purification cascades 1 and 2, which process 258,400 metric tons of BWH 3 annually, are listed in Table 4. It should be mentioned that the examinations carried out in this section have a conceptual character and are based on the experimental work from Section 3.1 and Section 3.2. The periodic renewal of membranes (lifetime 1–1.5 years [33,74,75]) and adsorption material (lifetime >5 years [76]) is included in the costing.

In purification cascade 1, 63% of the oligomeric hemicellulose in BWH 3 is converted to monomeric xylose and subsequently separated from potential inhibitors and concentrated to 200 g/L at a VR of 0.76. The resulting purified xylose stream of 7.8 t/h can be fermented, for example, to lactic acid [77], xylitol [78], or malic acid [55]. The advantage of this cascade is that no other input streams, such as additional chemicals, are needed. Nevertheless, a high wastewater quantity of 17 m³ per metric ton of xylose (dry matter = 100%) is produced. This high volume is mainly due to the significant water input during the organosolv pulping, as described in Section 2.1. In purification cascade 2, the hemicellulose in BWH 3 is separated from lignin and smaller molecules and concentrated to 200 g/L at a VR of 0.76. The resulting hemicellulose solution for further material use is 7.6 t/h. A 50 wt.% aqueous ethanol solution has proven to be suitable for removing lignin and other hydrophobic components from the polymeric adsorbent [35]. It was assumed that the adsorbent bed is recovered within 5 BVs of desorbate solution [34]. For this purpose, 16.2 t/h ethanol is needed, which is recovered by a rectification column with a rate of 99.5%. Hence, the required amount of fresh ethanol, including the losses during the desorption process, is 0.2 t/h. One metric ton of hemicellulose (dry matter = 100%) causes 25.3 m³ of wastewater for the same reason as explained above. 

The comparison between the two concepts shows that for purification cascade 1, more of the product xylose can be obtained with less wastewater. This is mainly due to the adsorption process within cascade 2 and the associated losses of sugars as well as higher requirements for auxiliary and operating materials.

#### 3.3.2. Energy Balance

Energy requirements and utility consumption of the purification cascades are shown in Table 5. Electricity for the pumping system of the adsorption and desorption process was calculated according to the hydraulic characteristics of the adsorbent material [80] and for the feed and recirculation pump for UF and NF according to Jönsson et al. [81]. The energy demand of the other process steps was determined using Aspen Plus^®^.

Within purification cascade 1, the hydrothermal treatment requires the total HPS, and the electricity demand is mainly for the NF process. In a comparative Aspen Plus^®^ simulation, where multiple-effect evaporation instead of NF realized the xylose concentration, the heat requirement is higher by a factor of 3.7 and the electricity demand is lower by a factor of 0.3. In purification cascade 2, the highest utility demands are LPS and CW, mainly for the recovery of ethanol after desorption but also for the adsorption and desorption process itself. The UF process consumed most of the electrical power.

For both purification cascades, the energy efficiency was determined, defined as the energy output in the purified product stream divided by the energy input, including all material streams as well as heat and electrical power demands. The calculations are based on the following estimations: (a) lower heating values (LHVs) of the input and the purified product streams were obtained from Aspen Plus^®^ and (b) process heat and electrical power are provided by a natural-gas-fired combined heat and power plant with a thermal efficiency of 58% and an electrical efficiency of 39% [82]. The energy efficiency for the purification of xylose in cascade 1 is 59% and for the purification of hemicellulose in cascade 2 is 26%. The significantly lower value of purification cascade 2 compared to purification cascade 1 can be explained by two circumstances. On the one hand, a higher energy input by the material stream ethanol (LHV = 26.7 MJ/kg) and the additional heat requirements for ethanol recovery. On the other hand, different retention values for furans, acetic acid, and lignin during NF and combined adsorption and UF. Meaning, the overall separation of the three components has a lower degree in purification cascade 2 compared to purification cascade 1. This results in a higher energetic loss but also a more purified product stream. However, by implementing an adsorption step in purification cascade 1, the separation could be improved. Furthermore, the targeted recovery of lignin could compensate for the associated decrease in energy efficiency as a by-product stream after desorption.

#### 3.3.3. Costing

FCI and annual costs for the two purification cascades are summarized in Table 6. The costs for the equipment are 2081 kEUR for purification cascade 1 and 2424 kEUR for purification cascade 2. The most expensive components for purification cascade 1 are the reactor for hydrothermal treatment (621 kEUR) and the NF (135 kEUR). For purification cascade 2, the costs are determined by the rectification column for ethanol recovery (638 kEUR), the adsorption columns (422 kEUR), and UF (50 kEUR). To calculate the FCI, a surcharge factor of 4.5 was used for equipment costs to account for a liquid treatment plant’s indirect and direct costs.

Since no costs are assumed for the BWH [55], the operating costs, mainly labor, account for a large share of the total costs. For purification cascade 1, this cost position is with 42% the most significant cost driver. On the other hand, due to the high energy demand, mainly LPS, of purification cascade 2, the consumption-linked costs have the highest share with 61%.

Specific production costs of the two purification cascades differ significantly (Table 7). The costs for the purified xylose solution in cascade 1, at 73.7 EUR/t, are almost halved compared to 135.1 EUR/t for producing purified hemicellulose solution in cascade 2. This difference is mainly, as already mentioned above, on the one hand, due to the higher product volume in cascade 1 compared to cascade 2, and on the other hand, due to more extensive process and energy requirements in cascade 2. However, identifying promising final products is essential as the next step for a complete economic assessment.

## 4. Conclusions

This study aimed to demonstrate and assess two purification cascades for the separation and valorization of hemicellulose from organosolv BWH. Purification cascade 1 consisted of a hydrothermal treatment of the BWH to hydrolyze remaining hemicellulose oligomers into xylose and NF to separate potential inhibitors (e.g., carboxylic acids and furanic compounds) as well as concentration. At a temperature of 180 °C, a residence time of 9 min, and the initial pH (2.6) of the BWH, 63% of the hemicellulose oligomers were hydrolytically split to xylose, and only minor furfural formation was observed, resulting in low sugar degradation. Appropriate process conditions for the NF process were a transmembrane pressure of 2 MPa and a temperature of 35 °C. Thereby, the hydrothermally treated BWH was concentrated to a VR of 0.8, resulting in an increase in xylose concentration by a factor of 4.8 and a reduction of the inhibitor-to-xylose ratio by 70%. Purification cascade 2 consisted of an adsorption process to remove foulants such as lignin from the BWH and UF to concentrate the hemicellulose while removing remaining lignin fragments and smaller molecules. After feeding 5 BVs of BWH to an adsorption column with a SEPABEADS SP700 fixed bed, 80% of the lignin was removed, associated with a hemicellulose recovery of 99%. Adsorption previous to the UF process increased the average permeate flux by a factor of 2.7 due to reduced fouling and decreased hemicellulose and lignin retention by a factor of 0.9 and 0.5, respectively. The concentration to a VR of 0.8 resulted in an increase in hemicellulose concentration by a factor of 4.2 and a reduction of the lignin-to-hemicellulose ratio by 43%.

Based on the findings of the experimental work, both purification cascades were simulated with Aspen Plus^®^, treating 258,400 metric tons of BWH annually (resulting from 50,000 dry metric tons of beechwood). Mass and energy balances showed that in cascade 1, 7.8 t/h of a purified xylose solution with a concentration of 200 g/L and, in cascade 2, 7.6 t/h of a purified hemicellulose solution with a concentration of 200 g/L could be gained. The total energy efficiency of the two cascades is 59% and 26%, respectively. Production costs for the purified xylose solution are 73.7 EUR/t, and for the purified hemicellulose solution, 135.1 EUR/t.

## Figures and Tables

**Table 1:** Composition of beechwood hydrolyzate 1 (BWH 1), 2 (BWH 2), and 3 (BWH 3), and physicochemical properties of oligomeric hemicellulose, glucose, xylose, 5-hydroxymethylfurfural (5-HMF), furfural, acetic acid, and lignin (MW: molecular weight).

Components	Concentration (g/L)			MW (g/mol)	pK_a_
	BWH 1	BWH 2	BWH 3		
Oligomeric hemicellulose	4.0	10.3	22.8	300–10,000 ^a,b^	12–13 ^c,d^
Glucose	0.6	1.7	2.8	180.16 ^e^	12.46 ^e^
Xylose	6.0	13.3	34.2	150.13 ^e^	12.14 ^e^
5-HMF	0.4	0.6	0.8	126.11 ^d^	n/a
Furfural	0.5	0.1	n.d.	96.08 ^d^	n/a
Acetic acid	4.1	3.8	3.9	60.05 ^e^	4.756 ^e^
Lignin	1.6	3.2	4.8	500–1000 ^a,b^	3–10 ^c,f^

^a^ [43]; ^b^ [48]; ^c^ [49]; ^d^ [31]; ^e^ [29]; ^f^ [50].

**Table 2:** Main assumptions for costing.

Parameter	Value	Unit
Average cost of capital	4	% p.a.
Assessment period	20	Years
Maintenance, repairs	5	% of FCI p.a. ^a^
Administration	20	% of labor costs p.a. ^a^
Insurance, uncertainties	1.5	% of FCI p.a. ^a^
Labor costs	28.5	EUR/h ^b^
UF/NF membrane	30	€/m² ^c^
Membrane housing	50	€/m² ^c^
Adsorbent material	5100	€/m³ ^c^

^a^ [60]; ^b^ [63]; ^c^ information from manufacturers and industry.

**Table 3:** Composition of the hydrothermally treated beechwood hydrolyzate 1 (HTBWH) at different process conditions.

	HTBWH 1.1	HTBWH 1.2	HTBWH 1.3
Pressure (MPa)	16.2	13.5	12.9
Temperature (°C)	180	174	180
pH (-)	2.6	1.7	1.7
Oligomeric hemicellulose (g/L)	1.5	0.0	0.0
Xylose (g/L)	8.2	10.3	9.2
Furfural (g/L)	0.7	n/a	1.3

**Table 4:** Mass balance, energy efficiency, and (production) costs of purification cascades 1 and 2 (assumed capacity: 258,400 metric tons of BWH 3 annually).

Purification Cascadeand Main Streams	Mass Input	Mass Output	Energy Efficiency	(Production) Costs
	[t/h]		[%]	[EUR/t]
Purification cascade 1				
Beechwood hydrolyzate 3	32.3 (10.3 MW)	0.0		
Wastewater	0.0	24.5		2.5 ^a^
Xylose solution (C = 200 g/L)	0.0	7.8 (8.0 MW)	59	73.7 ^c^
Purification cascade 2				
Beechwood hydrolyzate 3	32.3 (10.3 MW)	0.0		
Process water	12.2	0.0		0.15 ^a^
Ethanol	0.2 (1.7 MW)	0.0		550 ^b^
Wastewater	0.0	37.1		2.5 ^a^
Hemicellulose solution (C = 200 g/L)	0.0	7.6 (7.0 MW)	26	135.1 ^c^

^a^ [60]; ^b^ [79]; ^c^ calculated.

**Table 5:** Energy/utility requirements and costs of purification cascades 1 and 2.

Process Utility	Purification Cascade 1	Purification Cascade 2	Costs
	[MW]		
Electrical power	0.3	0.2	150 EUR/MWh ^a^
Cool water	0.0	7.2	0.043 EUR/t ^b^
Low-pressure steam	0.0	8.8	25 EUR/t ^b^
High-pressure steam	1.5	0.0	25 EUR/t ^b^

^a^ [83]; ^b^ [4].

**Table 6:** Investments and annual costs of purification cascades 1 and 2.

		Purification Cascade 1	Purification Cascade 2
Fixed-capital investments	[kEUR]	9415	10,962
Capital-linked costs	[kEUR/a]	696	809
Depreciation	[kEUR/a]	473	550
Interest	[kEUR/a]	223	259
Consumption-linked costs	[kEUR/a]	1543	5002
Raw Material	[kEUR/a]	-	-
Auxiliary and operating material	[kEUR/a]	51	1080
Energy Supply	[kEUR/a]	1001	3180
Disposal costs	[kEUR/a]	491	742
Operation-linked costs	[kEUR/a]	1906	1984
Labor Costs	[kEUR/a]	1436	1436
Maintenance	[kEUR/a]	471	548
Other costs	[kEUR/a]	428	452
Administration	[kEUR/a]	287	287
Insurance	[kEUR/a]	94	110
Uncertainties	[kEUR/a]	47	55

**Table 7:** Specific production costs of the purified xylose and hemicellulose solution.

		Purification Cascade 1	Purification Cascade 2
Capital-linked costs	[EUR/t_product_]	11.2	13.3
Consumption-linked costs	[EUR/t_product_]	24.9	81.9
Operation-linked costs	[EUR/t_product_]	30.7	32.5
Other costs	[EUR/t_product_]	6.9	7.4
Production costs	[EUR/t_product_]	73.7	135.1

## Data Availability

The data presented in this study are available on request from the corresponding author.
